# Altered Development of Mesencephalic Dopaminergic Neurons in SIDS: New Insights into Understanding Sudden Infant Death Pathogenesis

**DOI:** 10.3390/biomedicines9111534

**Published:** 2021-10-26

**Authors:** Anna Maria Lavezzi

**Affiliations:** “Lino Rossi” Research Center for the Study and Prevention of Unexpected Perinatal Death and SIDS, Department of Biomedical, Surgical and Dental Sciences, University of Milan, 20122 Milan, Italy; anna.lavezzi@unimi.it

**Keywords:** dopamine, midbrain, dopaminergic neurons, periaqueductal gray, smoking, substantia nigra, Sudden Infant Death Syndrome

## Abstract

Sudden infant death syndrome (SIDS) is defined as the unexpected sudden death of an infant under 1 year of age that remains unexplained after a thorough case investigation. The SIDS pathogenesis is still unknown; however, abnormalities in brain centers that control breathing and arousal from sleep, including dramatic changes in neurotransmitter levels, have been supposed in these deaths. This is the first study focusing on mesencephalic dopaminergic neurons, so far extensively studied only in animals and human neurological diseases, in SIDS. Dopaminergic structures in midbrain sections of a large series of sudden infant deaths (36 SIDS and 26 controls) were identified using polyclonal rabbit antibodies against tyrosine hydroxylase, the rate-limiting enzyme in catecholamine biosynthesis, and the dopamine transporter, a membrane protein specifically expressed in dopaminergic cells. Dopamine-immunolabeled neurons were observed concentrated in two specific structures: the pars compacta of the substantia nigra and in the subnucleus medialis of the periaqueductal gray matter. Anatomical and functional degenerations of dopaminergic neurons in these regions were observed in most SIDS cases but never in controls. These results indicate that dopamine depletion, which is already known to be linked especially to Parkinson’s disease, is strongly involved even in SIDS pathogenesis.

## 1. Introduction

Sudden infant death syndrome (SIDS) is defined as the death of an apparently healthy infant under 1 year of age, usually occurring during sleep, that remains unexplained after a thorough case investigation that includes a death scene assessment, an autopsy, and a clinical history review [1,2]. Little is known about SIDS pathogenesis except that it is probably caused by a combination of many circumstances and in particular, it affects babies who are vulnerable to various environmental stresses during a critical developmental period [3]. At present, the way that parents can reduce the risk of SIDS is by not smoking while pregnant and, after birth, by placing the baby on the back when asleep [4,5].

Recent SIDS research has been focused on the frequent occurrence of developmental defects of multiple nerve centers responsible for regulating breathing and sleep/wake transitions [6,7].

It is now supposed that also dramatic changes in neurotransmitter levels could be associated with awakening disorders, although their direct involvement in the pathogenetic mechanism of SIDS is not quite proven [8,9]. We recently reported an association between SIDS and decreased expression of orexin, a neurotransmitter synthesized by neurons of the lateral hypothalamus that take part in the modulation of the arousal from sleep, thus hypothesizing a role of orexin in promoting wakefulness [10]. Significantly lower levels of serotonin, a brain chemical involved in many functions, including the sleep-wake cycle regulation, have been observed in SIDS victims compared to the serotonin values found in infants who had died of known causes [11,12]. However, to date, no research has yet been carried out on the possible implication in the pathophysiology of SIDS of dopamine (DA) which, on the contrary, is one of the most-studied neurotransmitters in many neurodegenerative disorders, due to its fundamental role in neuromodulation of various biological processes, such as motor control, cognitive function and sleep–wake cycle regulation [13,14,15,16,17,18,19,20]. In particular, loss of midbrain dopaminergic (mDA) neurons, which are the main source of DA in the mammalian central nervous system, has been associated with alterations in arousal from sleep in patients with Parkinson’s disease [19].

The aim of this study was then to focus on the cytoarchitecture and functionality of the mDA system in a large series of SIDS cases to evaluate the possible involvement of developmental neuroanatomical and neurochemical anomalies of mDA neurons even in this syndrome. In particular, a specific immunohistochemical protocol designed to evaluate the neuronal expression of the tyrosine hydroxylase (TH), a marker of catecholaminergic neurons [21], and the dopamine transporter (DAT), a membrane-bound protein specifically expressed in the DA cells [22,23], was applied to 36 SIDS cases and 26 age-matched controls.

## 2. Materials and Methods

### 2.1. Study Subjects

The study included two groups of infants: a SIDS cohort and a control group.

#### 2.1.1. SIDS

The SIDS group consisted of 36 infants, 15 females and 21 males, aged from 4 to 30 postnatal weeks (mean age: 15.8 weeks). All cases were categorized as SIDS due to failure to determine the cause of death, after applying the 2006 guidelines provided by Italian Law n.31 “Regulations for Diagnostic Post Mortem Investigation in Victims of SIDS and Unexpected Fetal Death”. This law imposes in particular that all infants suspected of SIDS, who died suddenly within the first year of age, must undergo an in-depth anatomopathological examination, particularly of the autonomic nervous system.

#### 2.1.2. Controls

This group included 26 suddenly deceased infants, 10 females, and 16 males aged from 5 to 27 postnatal weeks (median age: 15.2 weeks) whose autopsies and medical history reviews established a precise cause of death. Specific diagnoses were: congenital heart disease (*n* = 10), severe bronchopneumonia (*n* = 6), pulmonary dysplasia (*n* = 3), myocarditis (*n* = 2), pericarditis (*n* = 1), sepsis (*n* = 1), malaria (*n* = 1), intraventricular hemorrhage (*n* = 1), and mucopolysaccharidosis type I (*n* = 1).

### 2.2. Information about Infant Death Circumstances and Risk Factors for SIDS

The Italian law requires that, for every case of sudden infant death, a complete maternal and infant medical history must be collected, including information about the main risk factors for SIDS (such as prone infant sleeping position, bedsharing, maternal smoking, alcohol and drug abuse before, during and after pregnancy, parental psychiatric illness, particulate air pollution exposure, etc.) [24].

Of the 62 newborns, 33 consisting of 30 SIDS and 3 controls, died during sleep, after being placed to sleep on their backs. They were not overdressed, there were no soft pillows or other objects in their cots and the infants’ faces were not covered. The other 20 infants died in different circumstances, mostly during the day. No illnesses were reported by the mothers before, during, and after pregnancy, including iron-deficiency anemia and diabetes. None of the mothers used illicit drugs or misused alcohol. Also, 25 of the 36 SIDS mothers (69%) declared to have been active smokers from before the onset of pregnancy, consuming more than 3 cigarettes/day. The remaining 11 mothers (31%) declared that they had never smoked cigarettes. In these cases, whenever possible, the mothers’ self-reported smoking was validated by quantification, from a lock of the victim’s hair, of cotinine, the main metabolite of nicotine, characterized by a long elimination half-life [25]. Only 5 of the 26 mothers in the control group (19%) admitted their smoking habit. These negative self-reported smoking statuses of the mothers were also validated by the cotinine test.

Table 1 summarizes the SIDS/Control case study, indicating the age-ranges, sex distribution, and the main information related to deaths.

### 2.3. Ethics Approval

Institutional review board approval was not required for this study since it meets the requirements laid down by the Italian Law n.31/2006, which states that all victims of unexpected perinatal death must be subjected to extensive autopsy investigations, according to appropriate consistent guidelines. Furthermore, the “Lino Rossi” Research Center of Milan University is the national referral center for the application of this law and the seat of the National Data Bank for the collection of all data relating to cases of sudden fetal and infant death, as established by article 3 of the aforementioned law. However, the parents of all infants included in the study provided written informed consent to autopsies, related research, and publication of the results.

### 2.4. Autopsy Protocol

In addition to the routine necropsy procedures, the autopsy protocol included an in-depth analysis of the autonomic nervous system and in particular of the brainstem, conducted according to the methodology described in depth in some of our previous articles [10,26,27]. This study focused on the midbrain. Our first goal was to determine the main mesencephalic centers where the DA neurons were located in the cases under study. Although three subtypes of DA neurons were identified in experimental studies on rodent midbrain (more specifically A9 neurons located in the substantia nigra pars compacta (SNpc), A10 neurons located in the ventral tegmental area (VTA), and A8 neurons located in the retrorubral field), to date, the studies conducted on humans, especially related to neurodegenerative disorders like Parkinson’s disease, only take into consideration the SNpc as a fundamental group of DA-secreting cells. Therefore, in order to identify and analyze the mesencephalic DA centers in the newborns under study, we firstly determined their localization in selected sections obtained from the superior to the inferior colliculus using the immunohistochemical technique described below, which was specifically designed to evaluate the neuronal expression of tyrosine hydroxylase (TH), a marker for catecholaminergic neurons, and since TH is also expressed in other catecholamine neurons, of the dopamine transporter (DAT), a membrane-bound protein specifically expressed in the DA cells [21,22,23]. The protocol then provided for the morphological examination of the identified mDA centers on Klüver-Barrera-stained slides.

### 2.5. Immunohistochemical Analysis of Tyrosine Hydroxylase (TH) and Dopamine Transporter (DAT)

In order to analyze the DA immunoreactive neurons, consecutive midbrain sections were alternatively incubated overnight at 4 °C with polyclonal rabbit anti-TH (Millipore, Milan, Italy), and anti-DAT (Santa Cruz Biotechnology, Segrate, Milan, Italy) primary antibodies, diluted in phosphate-buffered saline (PBS) at ratios of 1:500 and 1:300, respectively. Successively, for both markers, biotin-conjugated secondary antibody incubations (1:200, *cat #S-1000* Vector Laboratories, Burlingame, CA, USA) were then performed for 30 min at room temperature. After several washes in PBS, antibody complexes were localized using the avidin-biotin complex (ABC) system (Vectastain ABC Elite kit cat #PK6101, Vector Laboratories) followed by 3,3′-diaminobenzidine reaction. The sections were then counterstained with Mayer-hematoxylin for nuclei, coverslipped after dehydration in ascending concentrations of ethanol and cleared in xylene and observed under a Nikon Eclipse E800 light microscope (Nikon Corporation, Tokyo, Japan). The images of interest were captured using a Nikon Coolpix 8400 digital camera attached to the microscope. Negative controls were performed to evaluate the specificity of the immunohistochemical methods, by replacing the primary antibodies with PBS during the incubation in brain sections obtained from five cases for each group (SIDS and controls), during which staining did not occur.

#### Quantification of TH and DAT Immunohistochemical Results

In order to measure TH and DAT-immunopositivity, a qualitative rating system based on the examination of all the immunostained sections was adopted. A three-point scale was used to quantify the degree of immunopositivity in the specific areas. In short, the TH-and DAT immunoreactivity scores were classified as: mild (+)= few stained positive cells (<20%); moderate (++)= a number of positive cells ranging from 20% to 50%; marked (+++)= high number of darkly immunostained positive neurons (>50%).

### 2.6. Statistical Methods

All of the histological and immunohistochemical findings obtained using the above procedures were analyzed blindly by two independent pathologists. The evaluations obtained by each observer in relation to the different parameters were reported on a table on a case-by-case basis. After the mean values had been calculated, they were compared using the K Index (KI) in order to evaluate interobserver reproducibility. The Landis and Koch [28] methodology for interpreting K coefficient values was then applied, where 0 to 0.2 indicates slight agreement, 0.21 to 0.40 indicates fair agreement, 0.41 to 0.60 indicates moderate agreement, 0.61 to 0.80 indicates strong or substantial agreement, and 0.81 to 1.00 indicates very strong or almost perfect agreement (a value of 1.0 implying perfect agreement). Very satisfactory KI values (0.89 for histological evaluations and overall 0.90 for immunohistochemical evaluations) were obtained in this study. The statistical significance of the direct comparisons between groups was determined using the analysis of variance (ANOVA). Statistical calculations were carried out using the SPSS (statistical software package for social sciences). Differences were considered statistically significant if the *p* value was <0.05.

## 3. Results

### 3.1. Immunohistochemical Analysis of the Mesencephalic Dopaminergic (mDA) Neurons in SIDS and Controls

It is important to note that TH expression in both SIDS and controls was almost always in agreement with DAT expression, thus indicating that the catecholaminergic neurons identified by TH-immunohistochemistry were indeed dopaminergic.

#### 3.1.1. Control Group

**Figure 1:**
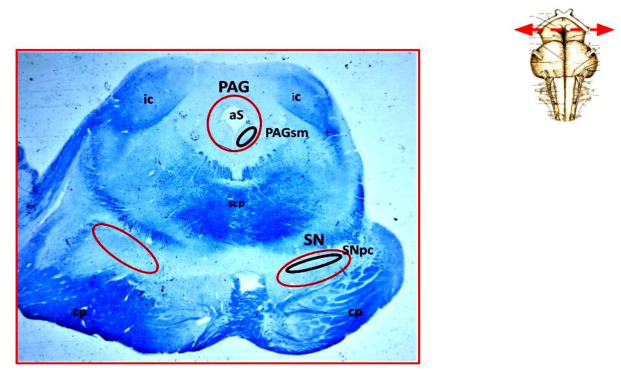
Photomicrograph of a transverse section of midbrain at the inferior colliculus level (at the top right, indication of the level corresponding to the section). The circled red area higher up, around the Sylvius aqueduct, indicates the location of the PAG; inside, circled in black, the location of the subnucleus medialis is represented; The bilateral circled areas lower in red indicate the localization of the substantia nigra; inside, the black circle circumscribes the pars compacta. Klüver-Barrera stain. Original magnification, 0.5×; aS = aqueduct of Sylvius; cp = cerebral peduncle; ic = inferior colliculus; PAG = periaqueductal gray; scpd = superior cerebellar peduncle decussation; PAGsm = periaqueductal gray subnucleus medialis; SN = substantia nigra; SNpc= substantia nigra pars compacta.

Notable levels of both TH and DAT markers were found in controls concentrated in perikarya and processes (dendrites and axons) of neurons located in two specific mesencephalic regions: (1) the pars compacta of the substantia nigra (SNpc), a structure located adjacent to the superior peduncles, and (2) a subpopulation of neurons in the anterolateral part of the periaqueductal gray (PAG), which is an area of the gray matter surrounding the cerebral aqueduct of Sylvius, namely the subnucleus medialis (PAGsm). Figure 1 shows the localization of these structures identified by means of immunohistochemistry.

**Figure 2:**
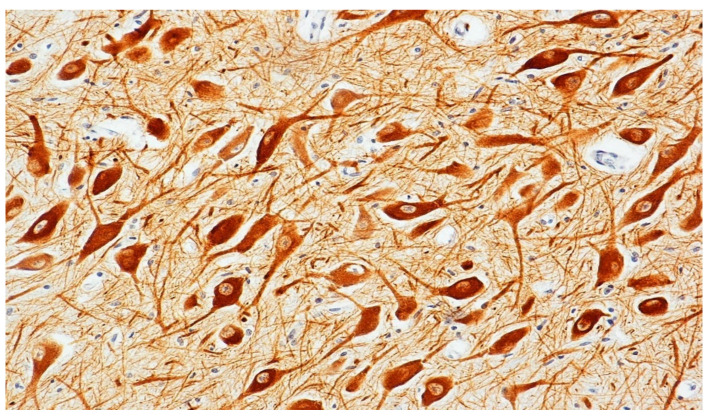
Tyrosine hydroxylase (TH) immunocytochemistry in a control case. Intensely dopamine-stained neuronal bodies and processes, graded as strong, are clearly visible. Original magnification, 20×.

In detail, high scores of mDA neurons (++/+++) were detected in 25 cases (Figure 2).

Only in one case the amount of immunopositive neurons in both SNpc and PAGsm was <20% (mild score/+).

#### 3.1.2. SIDS Group

**Figure 3:**
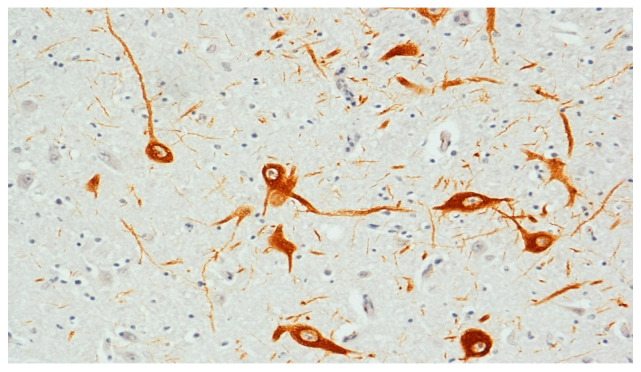
Tyrosine hydroxylase (TH) immunocytochemistry in a SIDS case graded as mild, due to the presence of few dopamine-stained neurons. Original magnification, 20×.

Moderate or strong TH/DAT immunopositivity in both SNpc and PAGsm structures was found only in 8 SIDS (22%). A significantly reduced number of DA-immunopositive neurons (mild score, +) was observed in the remaining 28 victims of this group (78%), all found dead in their sleep (Figure 3).

Table 2 summarizes the immunohistochemical results. The data presented demonstrate the high agreement between TH and DAT expression in both SIDS and control groups, and furthermore that their poor immunopositivity (mild score) was significantly more present in SIDS than in controls (*p* < 0.01).

### 3.2. Morphological Analysis of the Mesencephalic Dopaminergic (mDA) Neurons in SIDS and Controls

The histological examination was performed on midbrain sections adjacent to those used for the immunohistochemical analysis, stained with Klüver/Barrera. In most cases, the SNpc consisted of a dense streak of closely spaced neurons, dorsal to the other portion of the SN, that is, the pars reticulata, a relatively poor yet wider cell structure. Hypoplasia of the SNpc with a marked reduction in the number of neurons was observed in 23 SIDS cases (64%).

The PAGsm in control cases appeared as a wide cluster of large, sometimes elongated, neurons in the ventrolateral gray matter. A total of 20 SIDS cases with SNpc hypoplasia also showed neuronal loss in this subnucleus (hypoplasia).

### 3.3. Correlation between Morphological/Immunohistochemical Findings and Maternal Smoking

As shown in Table 3, a significant correlation was observed between SIDS, SNpc, and PAGsm hypoplasia, low TH/DAT immunostaining, and maternal smoking. In particular, infants who died of SIDS were more likely to have been exposed to maternal cigarette smoke than the controls (25 SIDS and 5 controls); 19 of the 25 SIDS victims and one of the 5 control subjects with smoking mothers showed mild TH/DAT-scores (+) in the SNpc and PAGsm neurons. Conversely, moderate/marked TH/DAT expression (++/+++) was detected in 23 of the 32 victims with non-smoker mothers (21 controls and 2 SIDS). SNpc and PAGsm hypoplasia was associated with maternal smoking exclusively in 15 SIDS cases.

### 3.4. Correlation between Morphological/Immunohistochemical Findings and Autoptic Gross Pathology in SIDS

In order to provide further information on SIDS pathogenesis, the autoptic albeit not pathognomonic findings, such as lung weight below normal age-related values, intrathoracic petechiae, and mild acute splenitis, were related to the above reported immunohistochemical and morphological results. A noteworthy association, although not significant, was observed in 5 SIDS cases between the presence of mild respiratory inflammation, hypoplasia of both SNpc and PAGsm and low TH/DAT immunostaining.

## 4. Discussion

Over the past decades, much research has been carried out on mDA neurons, generally focused on investigating their role in neurological and psychiatric disorders, such as Parkinson’s disease, schizophrenia, and attention deficit hyperactivity disorder [13,14,15,16,17,18,19,20].

The aim of this study was to extend the neuropathological knowledge of the DA system to the early stages of life, with the specific purpose of verifying the presence of DA developmental imbalances in SIDS and providing valuable insights into the pathogenetic mechanisms of this syndrome.

The identification of the mDA neurons in newborns was achieved by applying the immunohistochemistry for tyrosine hydroxylase (TH) and, since this enzyme is also expressed in other catecholaminergic neurons, for the dopamine transporter (DAT), a plasma membrane protein selectively expressed in DA neurons [21,22,23]. DAT in particular, is able to regulate the functional state of DA neurons by mediating the interaction between pre- and postsynaptic receptors in order to maintain the delicate balance between the quantity of DA synthesized, stored, and released [23]. As expected, DAT-labeling here detected, consistently agreed with the distribution of TH immunoreactivity.

The first notable result obtained in this study was that two well-delineated mesencephalic structures, the SNpc and the PAGsm, share the same enzymatic pathways involved in DA synthesis and release, while the second important result was the significant decrease in TH and DAT immunoreactivity observed in these specific neuronal centers in 78% of SIDS victims, all died during their sleep. The consequent depletion of intraneural DA stores and release may have induced alterations in respiratory control during the awakening phase, which is the most frequent condition in which SIDS occurs [6].

In fact, DA plays an important role, other than in the neuromodulation of motor control, emotions, cognitive functions, also in awakening from sleep [13,14,15,16,17,18,19,20]. It is known in fact that sleep disturbances occur in both schizophrenia and Parkinson’s disease (19,20). Dzirasa et al. [17] in particular, in an experimental study on mice demonstrated that DA promotes wakefulness, and that its depletion causes disorders of the awake state by diminishing the REM sleep. We can therefore assume that death in SIDS cases with mild DA immunopositivity occurred in the awakening phase.

Noteworthy was also the relationship highlighted between hypoimmunoreactivity of mDA neurons in SIDS and maternal smoking behavior.

It is common knowledge that exposure to nicotine during pregnancy through maternal smoking causes, in addition to adverse effects on the placenta, serious health problems for unborn babies, such as preterm birth, low birth weight, stillbirth, and increasing risk of SIDS [29,30,31,32]. The toxic effects of prenatal exposure to nicotine and carbon monoxide (CO), one of its gaseous combustion products, are well documented. Both substances are able to readily cross the placenta by passive diffusion, reaching concentrations in the fetal compartment that are generally 15% higher than maternal levels [32]. In the fetal bloodstream, carboxyhemoglobin, the product of a reaction between CO and hemoglobin, is unable to release oxygen into fetal tissues causing hypoxia, especially in the most susceptible organs, including the brain. Moreover, nicotine, which is one of the few fat-soluble substances that can easily pass through the blood-brain barrier, can alter neuronal migration, proliferation, and differentiation thus leading to dysregulation of brain neurodevelopment including disruptions in neurotransmitter systems [33,34,35,36,37,38,39,40,41]. In particular, Muneoka et al. [39] demonstrated in rats that nicotine absorption in utero induces disturbances in the DA system and causes a predisposition to diseases related to DA dysfunctions. Accordingly, Oliff et al. and Keller et al., [40,41] in other experimental studies, showed that catecholamine systems are particularly sensitive to perinatal nicotine exposure resulting in a reduction in DA release.

We, therefore, may believe that the morphological and functional alterations of the mDA centers highlighted in most SIDS cases in this study may be caused by maternal smoking in pregnancy. The chemicals in cigarette smoke may have reduced during pregnancy the number of neurons and the expressiveness of functional markers involved in DA synthesis in specific mesencephalic structures, that is, the SNpc and the PAGsm, during fetal nervous system development. Given that DAexerts an important control over the various stages of sleep, the damage produced by these defects can cause after birth serious breathing difficulties, especially in the awakening phase up to breathing cessation.

In conclusion, by highlighting the loss of SNpc and PAGsm neurons frequently associated with reduction of TH and DAT immunoexpression, and the disruption of the DA system homeostasis induced by the smoke absorption in pregnancy, this study offers new insights into understanding the complex and multifaceted pathological process that leads to SIDS.

### Future Directions

Further research is required to get a clearer picture of the effect of nicotine on developing mDA system in SIDS. It is essential to enhance the knowledge on this issue by investigating the gene expression profile of mDA neurons in SIDS, as has already occurred with other pathologies [42,43,44]. The repertoire of genes expressed in mDA neurons could provide further crucial information on their physiology and on the mechanisms of specific dysfunctions occurring in SIDS.

## Figures and Tables

**Table 1:** Demographic Data of the Individuals Included in this Study.

	SIDS	CONTROLS
Number of cases	36	26
Sex, male/female	21/15	16/10
Postnatal age in weeks, range (mean)	4–30 (15.8)	5–27 (15.0)
Died during sleep	30	3
maternal smoking, yes/no	25/11	5/21

**Table 2:** Comparison of TH and DAT Immunoreactivity in the midbrain of SIDS and Controls.

Cases	TH						DAT					
	SNpc			PAGsm			SNpc			PAGsm		
	+	++	+++	+	++	+++	+	++	+++	+	++	+++
SIDS (36)	28	7	1	28	8	0	28	4	4	28	5	3
	(78%)	(19%)	(3%)	(78%)	(22%)	(0%)	(78%)	(11%)	(11%)	(78%)	(14%)	(8%)
CONTROLS (26)	1	10	15	1	13	12	1	5	20	1	11	14
	(4%)	(38%)	(58%)	(4%)	(50%)	(46%)	(4%)	(19%)	(77%)	(4%)	(42%)	(54%)

The categorical data are expressed as number of cases and percentages. DAT: dopamine transporter; PAGsm: periaqueductal grey subnucleus medialis; SNpc: substantia nigra pars compacta; TH: tyrosine hydroxylase. + = mild immunopositivity; ++ = moderate immunopositivity; +++ = marked immunopositivity.

**Table 3:** Correlation of results with maternal smoking.

CASES		MATERNAL SMOKING Yes ^a^ No
SIDS (n.36)		25 (69%) 11 (31%)
SNpc cytoarchitecture	normal (n.13)	4 (11%) 9 (25%)
	hypoplasia (n.23)	21 (58%) 2 (6%)
PAGsm cytoarchitecture	normal (n.16)	6 (17%) 10 (27%)
	hypoplasia (n.20)	19 (53%) 1 (3%)
TH/DAT immunoexpression	score ++/+++ (n.8)	6 (17%) 2 (6%)
	score + (n.28)	19 (53%) 9 (25%)
CONTROLS (n.26)		5 (19%) 21 (69%)
SNpc ytoarchitecture	normal (n.26)	5 (19%) 21 (81%)
	hypoplasia (n.0)	0 (0%) 0 (0%)
PAGsm cytoarchitecture	normal (n.26)	5 (19%) 21 (81%)
	hypoplasia (n.0)	0 (0%) 0 (0%)
TH/DAT immunoexpression	score ++/+++ (n.25)	4 (15%) 21 (81%)
	score + (n.1)	1 (4%) 0 (0%)

Categorical data are expressed as number of cases and percentages. ^a^ Statistical significance of SIDS and Control group values in relation to maternal smoking *p* < 0.01. DAT: dopamine transporter; PAGsm: periaqueductal grey subnucleus medialis; SNpc: substantia nigra pars compacta; TH: tyrosine hydroxylase.

## Data Availability

The data presented in this study are available on request from the author.
